# Using Administrative Data to Identify Factors Associated with Healthy Development After Experiencing Household Challenge Adversity in Early Childhood

**DOI:** 10.23889/ijpds.v9i5.2803

**Published:** 2024-09-18

**Authors:** Anita Durksen, Wanda Phillips-Beck, Jon McGavock, Tracie Afifi, Marni Brownell, Nathan Nickel

**Affiliations:** 1 University of Manitoba; 2 Manitoba Centre for Health Policy; 3 First Nations Health and Social Secretariat of Manitoba

This study aimed to identify factors associated with healthy development after experiencing household challenge adversity in early childhood.

A cohort of 42,505 children born in Manitoba, Canada was created using linkable health, social, and education administrative data.  Children were divided into 5 groups according to whether they experienced adversity between birth and four years of age in the following categories: 1) having a parent diagnosed with a mental health disorder, 2) having a parent diagnosed with a substance use disorder, 3) having a parent who received income assistance for at least 2 months, 4) experiencing at least one of the 3 adversities, and 5) having none of the adversities listed. For each group, multiple linear regression models were computed to determine the association of the independent variables (age, sex, birth order, team parenting, neighbourhood-level socio-economic status, parent high school completion, being an established resident in Canada, not having a major illness, residential stability, program participation, and connectedness to health care services) on child development, represented by their Early Development Instrument scores.

In each group, factors associated with healthy development included at minimum: older age, being female, being firstborn, team parenting, higher neighbourhood-level socio-economic status, parent high school completion, being an established resident in Canada, and not having a major illness. Several of these factors are associated with increased access to material resources. As such, policies that facilitate the delivery of resources to lower-income families and neighbourhoods with lower socioeconomic status could translate into improved developmental outcomes for children.

